# Estimating life expectancy adjusted by self-rated health status in the United States: national health interview survey linked to the mortality

**DOI:** 10.1186/s12889-021-12332-0

**Published:** 2022-01-20

**Authors:** Hyunsoon Cho, Zhuoqiao Wang, K. Robin Yabroff, Benmei Liu, Timothy McNeel, Eric J. Feuer, Angela B. Mariotto

**Affiliations:** 1 Department of Cancer Control and Population Health, Graduate School of Cancer Science and Policy, National Cancer Center, 323 Ilsan-ro, Ilsandong-gu, Goyang-si, Gyeonggi-do 10408 Republic of Korea; 2 Information Management Services Inc., Calverton, MD USA; 3 Surveillance and Health Equity Science, American Cancer Society, Atlanta, GA USA; 4 Division of Cancer Control and Population Sciences, National Cancer Institute, Bethesda, MD USA

**Keywords:** Self-rated health status, Life expectancy, Survival, Mortality, health survey, National Health Interview Survey

## Abstract

**Background:**

Life expectancy is increasingly incorporated in evidence-based screening and treatment guidelines to facilitate patient-centered clinical decision-making. However, life expectancy estimates from standard life tables do not account for health status, an important prognostic factor for premature death. This study aims to address this research gap and develop life tables incorporating the health status of adults in the United States.

**Methods:**

Data from the National Health Interview Survey (1986–2004) linked to mortality follow-up through to 2006 (age ≥ 40, *n* = 729,531) were used to develop life tables. The impact of self-rated health (excellent, very good, good, fair, poor) on survival was quantified in 5-year age groups, incorporating complex survey design and weights. Life expectancies were estimated by extrapolating the modeled survival probabilities.

**Results:**

Life expectancies incorporating health status differed substantially from standard US life tables and by health status. Poor self-rated health more significantly affected the survival of younger compared to older individuals, resulting in substantial decreases in life expectancy. At age 40 years, hazards of dying for white men who reported poor vs. excellent health was 8.5 (95% CI: 7.0,10.3) times greater, resulting in a 23-year difference in life expectancy (poor vs. excellent: 22 vs. 45), while at age 80 years, the hazards ratio was 2.4 (95% CI: 2.1, 2.8) and life expectancy difference was 5 years (5 vs. 10). Relative to the US general population, life expectancies of adults (age < 65) with poor health were approximately 5–15 years shorter.

**Conclusions:**

Considerable shortage in life expectancy due to poor self-rated health existed. The life table developed can be helpful by including a patient perspective on their health and be used in conjunction with other predictive models in clinical decision making, particularly for younger adults in poor health, for whom life tables including comorbid conditions are limited.

**Supplementary Information:**

The online version contains supplementary material available at 10.1186/s12889-021-12332-0.

## Background

Life expectancy is the most common summary measure of mortality used to describe the overall health status of a population. It is increasingly incorporated in evidence-based screening and treatment guidelines to ensure that patients with limited life expectancy exposed to the potential harms of screening and treatment live long enough to experience the potential benefits [1–4]. For example, the survival benefit from the early detection and effective treatment of colorectal or breast cancer may only occur 5 years after the screening test [2]. An individual with a shorter life expectancy (i.e., < 5 years) would not live long enough to benefit from early detection, yet such an individual will be exposed to the psychological distress of undergoing a cancer screening and diagnosis, complications from biopsies, and the risk of overtreatment. Thus, providing accurate life expectancy estimates that incorporate an individual’s health status is important to identify individuals who may not benefit from screening or treatment and to include discussions on the pros and cons of the intervention in the clinical decision-making process.

Predictions of life expectancies for the average person are usually obtained from national life tables stratified by age, sex, and race [5]. However, the survival experience of the average person does not fully account for other important prognostic factors of premature death, such as health status, comorbidity, and various behavioral and socioeconomic risk factors. Previous studies have developed tools to estimate life expectancy using additional characteristics, such as comorbidities, [6–10] smoking status, [11] and socioeconomic factors [12–14]. However, these tools can have limited applications. For example, Medicare claim data life tables by comorbidity status have only been calculated for the elderly population (65 years and older) based on the comorbidities that were treated and reimbursed. However, such data may not be readily available at the point of care. Moreover, little research has been conducted in the younger population, where individuals with high comorbidity burden or poor health status are likely to have a very different life expectancy than an average individual of the same age.

Studies have shown that self-rated health is a good predictor of mortality, [15–31] even after controlling for various objective health measurements (e.g., disease conditions, health risk behaviors such as smoking and exercise). Self-rated health is a summary measure that represents an individual’s evaluation of his/her own health, and captures the full array of illnesses a person has, possible disease symptoms yet to be diagnosed or treated, as well as the severity of current illness. Importantly, self-rated health consists of a single, easily understood and answered, and well-validated question that a physician or other health professional can query a patient, and it does not require comprehensive access to medical records or claim data [29]. However, studies estimating life tables solely by self-rated health in US adults are limited. Moreover, life expectancy by self-rated health status for the US adults compared to the US general population has not been estimated.

This study aims to address this research gap and develop life tables, and life expectancy estimates that incorporate self–rated health in an exceptionally large national sample of adults in the United States.

## Methods

### Data sources and study population

This study used data from the 1986–2004 public use National Health Interview Survey (NHIS) linked to mortality follow-up through to December 31, 2006 [32]. The NHIS is an annual in-person household survey of the civilian noninstitutionalized population of the United States conducted by the National Center for Health Statistics (NCHS). The civilian noninstitutional population includes adults who do not live in institutions (for example, correctional facilities, long-term care hospitals, and nursing homes) and who are not on active duty in the Armed Forces [33]. Response rates were well over 80% throughout the 19 years of NHIS data included in this study. The NCHS provides files to allow the linkage of NHIS participants to the National Death Index through December 31, 2006 for vital status information. The date of death was available in quarterly months for those who died; in addition, based on NCHS guidance, individuals that were not linked to the National Death Index were presumed to be alive on December 31, 2006. Adults younger than 40 years were excluded because 98.2% of those individuals were alive at the end of follow-up and would be considered censored cases in the survival analysis.

### Measures

The self-ratings of health status were based on responses to the question in the NHIS Family Core component: “Would you say your health in general is excellent, very good, good, fair, or poor?” All 5 health status categories were evaluated in this study. Demographic characteristics such as age, sex, and race (White, Black, other/multiple, unknown) were also considered.

### Survival estimation

Survival time was measured from the NHIS interview (month) to death (quarter) or end of follow-up in 2006. We estimated age-specific survival curves stratified by sex. We fit discrete time proportional hazards models by sex and 5-year age groups (i.e., 40–44, 45–49, 50–54, 55–59, 60–64, 65–69, 70–74, 75–79, 80–84, 85+). The following covariates were included in the model: health status (i.e., excellent, very good, good, fair, poor), race (i.e., white, black, other), survey year, and age, in a linear term within each age group. As proxy responses were accepted for family members that were not at home during the interview, reporting of self-rated health status (i.e., self, proxy) was also adjusted in the analysis. The models were fitted separately for each age-group to account for changes in baseline hazards by age and to more accurately characterize discrepancies in mortality caused by health status in each age group. The discrete time complementary log-log model, [34–36] a discrete analog of the Cox proportional hazards model, was employed for the proportional hazards models. All statistical analyses were conducted using SAS 9.3 (SAS Institute, Cary NC, USA) and SAS-callable SUDAAN [37] to account for the complex NHIS survey design and weights using SAS PROC SURVEYLOGISTIC. Details on model formulation and the SAS programs are provided in the supplementary materials. Sensitivity analyses were conducted for different model assumptions, and they too are reported in the supplemental materials. As approximately 36% reports were from family members as opposed to being self-reported, the effects of the proxy reporting of health status on hazards of death was also assessed.

### Life expectancy calculation

Life expectancy calculations require extrapolation of survival models beyond observed data. Although we had long-term follow-up, with the maximum follow-up at 21 years (Median: 9.8, IQR: 5.5, 14.7), annual survival probabilities beyond the follow-up period need to be estimated. Similar to other studies, the best matching US life table approach was used [6, 7] to extrapolate survival post-follow-up. For example, the life table estimated from the survival model for individuals aged 60 years stops at age 81 years. To extrapolate survival beyond age 81, we compared the modeled survival probabilities of a 60-year-old white man with excellent health to the age-specific US life tables of white men between ages 40 and 70, and identified the US life table of a 51-year-old white man as the best matching survival experience. Then, we defined a 51-year old as health adjusted age for a 60-year-old white man with excellent health. This best-matching life table was then used to calculate life expectancy (i.e., life expectancy at age 51 from the US life table is the health status adjusted life expectancy for a 60-year-old white man with excellent health). In this study, we used decennial 2000 US life tables by sex and race because they correspond closely to the study period [38, 39]. More details on these methods are provided in the supplemental materials (Supplemental Fig. 1).

## Results

### Demographics and health status

**Fig. 1:**
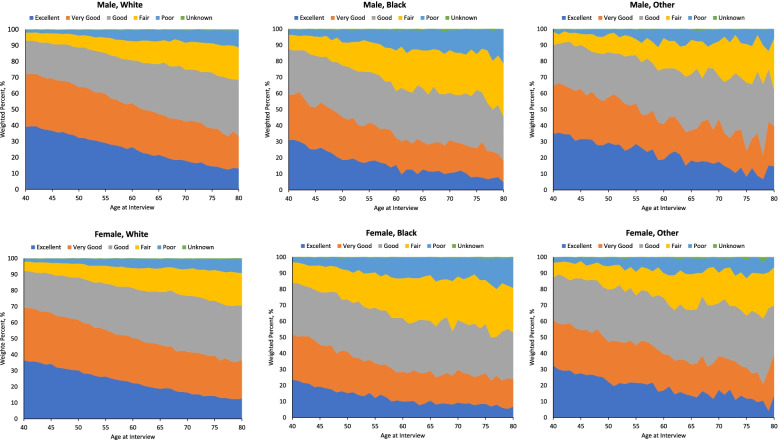
Prevalence of health status across age, NHIS survey years 1986–2004 combined

The study population consisted of 729,531 US adult respondents, aged 40 years and older at the time of the survey. The study population was predominately white (85.2%) and approximately half were women (53.4%) (Table 1). Health status ranged from 24.9% *excellent*, 28% *very good*, 28.6% *good*, 12.7% *fair*, to 5.4% *poor* (Table 1). The distribution of self-rated health status differed by age at the interview, with *excellent* health reported proportionately lower in the older participants, while *fair* to *poor* health status was higher in the older participants (Fig. 1). Health ratings were similar in men and women, though men were slightly more likely to rate their health as “excellent” relative to women in younger participants: for example, the proportion of those who rated their health as *excellent* among men and women at age 40 were: 39.2 and 36.4% in whites and 30.9 and 23.5% in blacks, respectively.

**Table 1:** Characteristics of study population: US adult respondents, age 40 years and older at the time of the interview, National Health Interview Survey, 1986–2004, with mortality follow-up through 2006

Variable		Sample size, n^a^	Wt^b^ %
Age at interview	40–44	136,139	18.9
	45–49	116,846	16.2
	50–54	98,198	13.6
	55–59	83,240	11.4
	60–64	75,184	10.1
	65–69	69,394	9.2
	70–74	58,886	7.9
	75–79	44,336	6.1
	80–84	28,184	3.9
	85+	19,124	2.7
Sex	Male	333,588	46.6
	Female	395,943	53.4
Race	White	601,184	85.2
	Black	90,655	9.9
	Other / multiple	34,368	4.5
	Unknown	3324	0.4
Health Status^c^	Excellent	178,179	24.9
	Very good	198,346	28.0
	Good	211,181	28.6
	Fair	97,056	12.7
	Poor	42,141	5.4
	Unknown	2628	0.3
Survival status	Dead	165,616	20.5
	Alive	563,915	79.5

^a^Sample size, total sample size was 729,531

^b^Weighted percent

^c^Includes health status reported by self (63.4%), proxy (36%) and missing (0.6%) reporting status information

### Impact of health status on survival

**Fig. 2:**
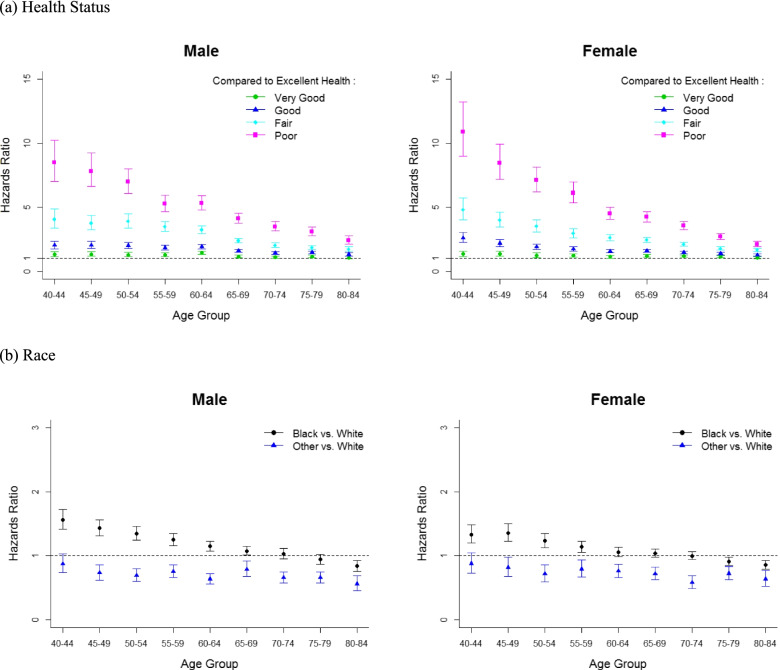
Impact of self-rated health and race on life expectancy by age groups. Hazards ratios for death and 95% confidence intervals were presented: (**a**) Impact of self-rated health status in white persons (reference is excellent health status at each age group), (**b**) Impact of race

Individuals reporting poor and fair health had a much higher risk of death compared to individuals reporting excellent health, and the difference in risk by health status was substantially higher in younger than older people (Fig. 2a). For example, among white men aged 40 years, those reporting *poor* health had 8.5 times (95% CI: 7.0, 10.3) greater risk of death compared with those reporting *excellent* health; whereas among white men aged 80 years, risk of death was 2.4 times higher (95% CI: 2.1, 2.8) for poor health compared to excellent health. In white women, comparisons of *poor* and *excellent* health correspond to 10.9 times (95% CI: 9.0, 13.2) greater risk of death at age 40 years and 2.1 times (95% CI: 1.9, 2.4) at age 80 years. Risk of death for those with *very good* and *good health*, compared to those in *excellent* health at the same chronological age, were similar across age groups. Figure 2b shows the effect of race on risk of death. Relative to white participants, black participants had a greater risk of death at younger ages but had similar or lower risk at older ages. At younger ages, the impact of health status on survival and life expectancy was greater in black compared to whites. While other races had a lower risk of death compared to whites overall.

**Fig. 3:**
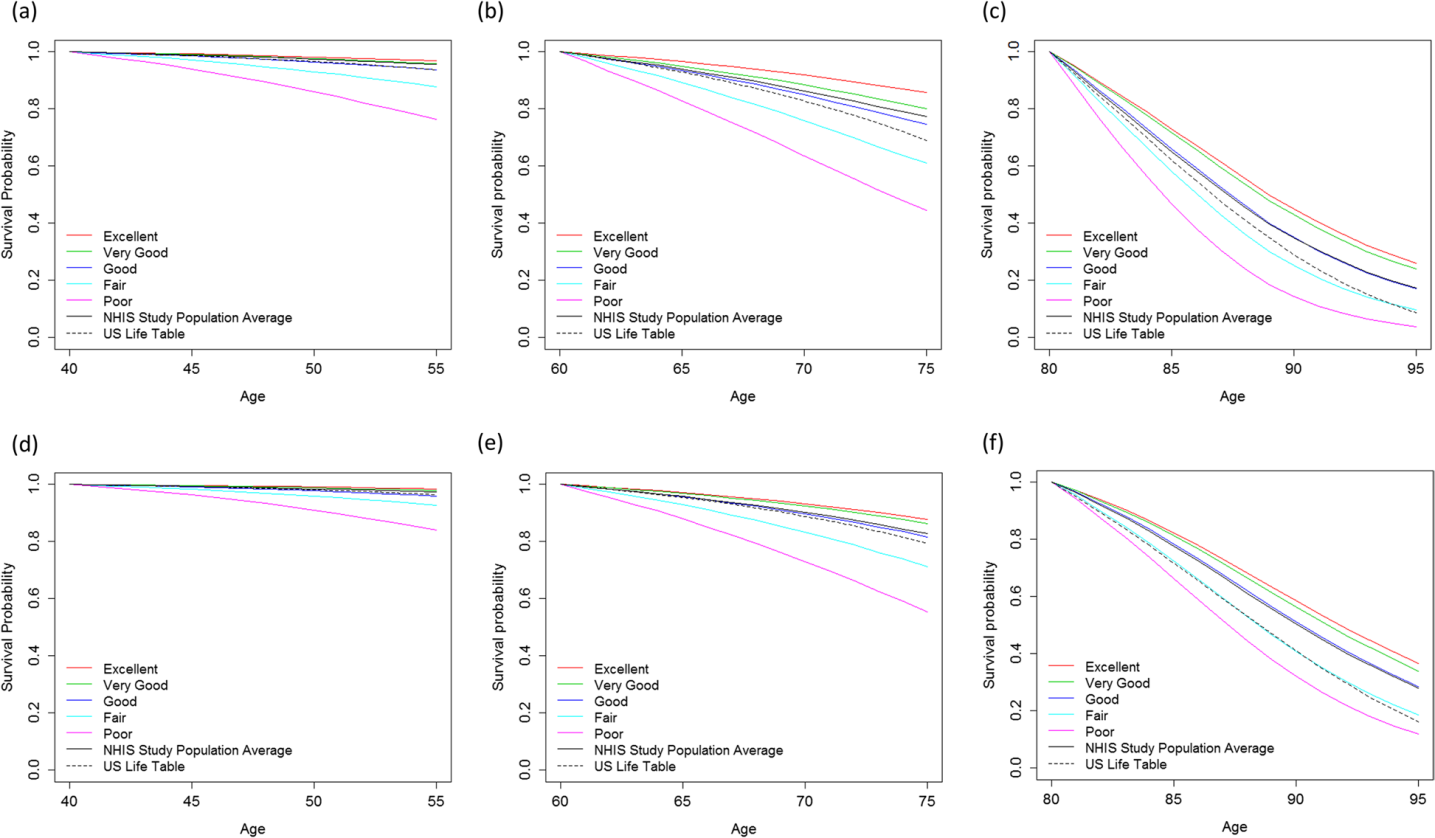
Estimated survival by self-rated health status at the time of interview at ages 40, 60 and 80 years. Note that smoothed lines are plotted for better visualization and results are same as the step function. (**a**) Age at interview 40 years, white male, self-respondent, (**b**) Age at interview 60 years, white male, self-respondent, (**c**) Age at interview 80 years, white male, self-respondent, (**d**) Age at interview 40 years, white female, self-respondent, (**e**) Age at interview 60 years, white female, selfrespondent, (**f**) Age at interview 80 years, white female, self-respondent

Estimated survival varied greatly by age and health status among white participants (Fig. 3), while the results were similar for blacks and other races (data not shown). There is almost no or little difference in survival among health status at a younger age. However, the small change in the survival probability will result in a large difference in life expectancy. The effect of health status on survival is strong for middle to old age. At older age, the effect of health status becomes smaller again. In other words, life expectancy in higher ages has less variability.

The survival experiences of individuals with favorable health status were generally similar (i.e., *excellent, very good,* and *good* health in younger participants; *excellent* and *very good* health in the elderly). As expected, survival probabilities were dramatically lower in older participants. Survival was close to 100% for individuals aged 40 to 55 with *excellent* to *good* health. Indeed, the survival estimated from the US life tables is generally close to the survival for people in *good* health. Individuals of all ages who reported *fair* and *poor* health had worse survival estimates relative to the US general population.

Notably, older individuals’ survival was higher in the NHIS study population compared to the US general population. This is likely because the NHIS sample did not include institutionalized individuals, who likely have a higher mortality risk. For example, 5-year (and 10-year) survival of the average white woman conditional on surviving at age 80 is 78% (50%) in the NHIS population, while it is 72% (41%) in the US general population. As such, at age 80, the survival experience of the US general population was close to individuals who reported fair health in the NHIS.

The sensitivity analysis used different models and found that the estimated survival outcomes were similar regardless of model selection (Supplemental Fig. 2). Compared to self-report, family reported (proxy-report) health status showed lower mortality risk (risk of death) in reported excellent health at all ages and higher mortality risk in individuals with poor health at older ages. However, the magnitudes were minor and the effects were not significant in some age groups (Supplemental Fig. 3).

### Life expectancy adjusted by health status

Life expectancy varied considerably by self-rated health status. When compared to the US general population matched by age, sex, and race, the life expectancies of those in *excellent* and *very good* health were longer, while of individuals with *fair* and *poor* health were shorter (Table 2). The remaining life expectancy at 60 males in the US general population was 20 for whites, 17 for blacks, 24 for others. In contrast, when considering health status, the remaining life expectancies varied substantially; those were 27 for *excellent*, 24 for *very good*, 21 for *good*, 17 for *fair*, 14 for *poor* health status in whites; 28, 25, 22, 17, 12 for blacks; 31, 28, 25, 20, 16 for other races. Variabilities between life expectancy by health status and general population average were more substantial in blacks than those in other races. The results were similar in the females; however, there existed a larger variability by health status. Discrepancies in life expectancy by health status were greater in younger ages. For example, life expectancies of white men in *excellent* health versus *poor* health is 45 versus 22 years (i.e., 23-year difference) at age 40, whereas it is 10 versus 5 years (i.e., 5-year difference) at age 80. In other words, at age 40, life expectancy (the differences in life expectancy relative to the US population average) for *excellent, very good, good, fair, poor* were 45 (8),42 (5), 37 (0), 29 (− 8), 22 (− 15); their remaining life vary greatly from 22 to 45, and the difference with US average was greater up to 15 years. In contrast, those were 10 (3), 10 (2), 8 (1), 7 (0), 5 (− 2) at age 80, showing, at older ages, less variability in life expectancy. Life expectancy highly depends on age, and in older ages, the effect of other factors (such as self-rated health), will become less distinctive.

**Table 2:**
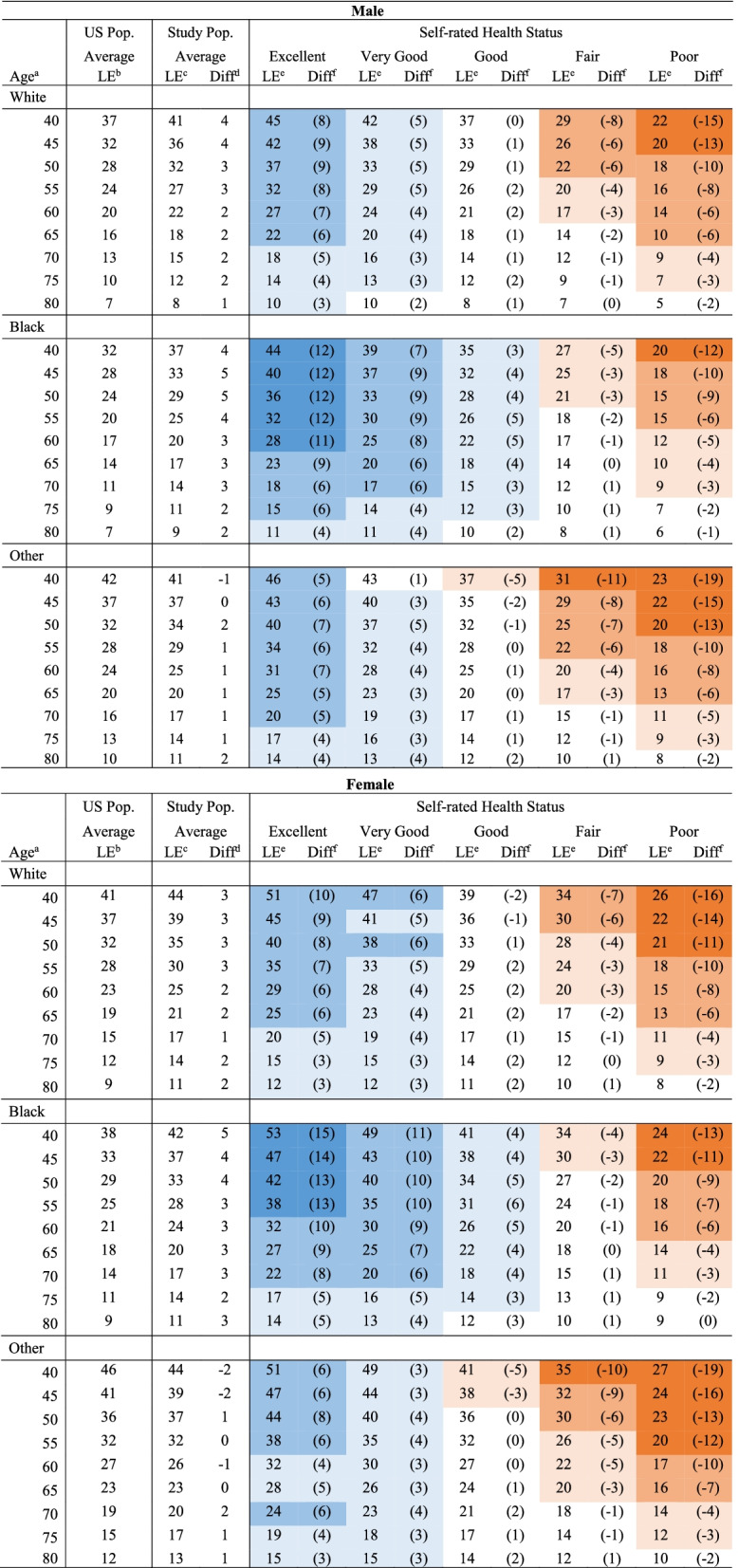
Life expectancy adjusted by self-rated health status compared with the life expectancies of the US average population

For an easy representation of the results, differences in life expectancies from 3 to 5 is shaded in light colors, 6 to 10 is shaded in medium colors, and more than 10 is shaded in dark colors. Blue colors represent greater and orange colors represent shorter life expectancies relative to the US average population

^a^Chronological age

^b^Life expectancy obtained from 2000 US decennial life table

^c^Average life expectancy of study population estimated from the model

^d^Differences in life expectancies calculated as the average life expectancy in the study population minus life expectancy in the average US population (2000 US life table)

^e^Life expectancy adjusted by self-rated health status estimated from the model

^f^Differences in life expectancies calculated as life expectancy adjusted by health status minus life expectancy in the average US population (2000 US life table)

The life expectancies of the NHIS study population were closer to reported good health status rates in all ages (Table 2). Life expectancies of the average US general population in the elderly are shorter, particularly in blacks, than those estimated from *good* health status in the NHIS population. For example, in blacks, the average life expectancy of the US general population is 4 years shorter at age 65 and 3 years shorter at age 70 (Table 2).

## Discussion

This study developed life tables and estimated the life expectancy by self-rated health for men and women in the United States using a large population-based dataset. Life expectancy was found to vary considerably by health status. Consistent with previous studies, [25, 27] self-rated health was found to be a reliable predictor of long-term survival, and individuals who rated their health as excellent had higher survival and life expectancy estimates than individuals with poor or fair health.

More importantly, self-rated health was found to have a greater effect on survival and life expectancy at younger ages compared to older ages. Younger people with poor health had a significantly shorter life expectancy compared to average persons at the same age. The life tables by health status presented here are an improvement compared to the national average and are particularly important for the understudied younger populations because clinical decision-making and patient-centered care may involve an assessment of benefits and harms for a younger person similar to that for an elderly person. Therefore, this study, by providing such data, represents an important contribution to the scarce literature on individuals younger than 66 years old.

This study builds upon a growing body of research addressing health status and life expectancy for use in clinical decision-making [6–9, 24, 25, 40]. Some studies incorporated utility scores, a composite index, for example, the Health Utility Index in Canada [38, 39], for the health state classifications or health-related quality of life in life expectancy evaluations. Other studies estimated healthy life expectancy [41–43], defined as the number of years that a person at a given age (often calculated at birth) can expect to live in good health (or full health), and utilized as a summary metric for mortality. In contrast, our life expectancy estimates adjusted for self-reported health status provide the average remaining life expectancy at specific health status reported. The measurement is based on a single question regarding health status, which is easily asked and answered, and essential demographic information. Meanwhile, prior works that have created life tables taking into account comorbidity status have been estimated from Medicare claims linked to the Surveillance Epidemiology and End Results (SEER) data for individuals aged 66 years and older [6, 7, 9]. However, these life tables are limited by the fact that they are unavailable for ages below 66, based on comorbidities measured from claims data, which only identify treated comorbidities, and they do not account for other factors that may affect health and mortality. Similarly, even prognostic indices and prediction models utilizing health status are often tailored to the elderly population and require additional information (i.e., existing illnesses) other than general health status to formulate a prognostic index to predict mortality. This study provides an extensive set of life tables by race, sex, and self-reported health status; it included participants aged between 40 and 64. Moreover, self-rated health has been shown in some studies to be a more accurate measure of health status than objective measures, [30, 44] as it reflects an individual’s perception of health and likely captures the full array of illness in a particular person.

We used an age-specific discrete time proportional hazards model to minimize assumptions and capture changes in the baseline hazards of each age group. The models were stratified by 5 years age groups because we did not want to assume proportionally in the baseline hazard age for different age groups, e.g., 40–44 and 60–64. However, the proportionality assumption was only used for ages within an age group. The proportional hazards assumption for other covariates was checked. This model allowed us to account for variation in both baseline hazards and the effect of health status on survival by age group. Therefore, the model enabled us to characterize the impact of health status in each age group and compare life expectancy adjusted for health status with the US life table estimates of the same age. Other models could be considered depending on the assumptions regarding the baseline hazards and the effect of health status across age groups. An overview of our sensitivity analyses using different models is provided in the Supplemental Material. Estimated survival outcomes were similar across various approaches.

There were several limitations to this study. The average survival and life expectancy of the NHIS study population was close to those who reported *good* health. However, as the NHIS population consists only of noninstitutionalized individuals, our average survival and life expectancy estimates were slightly higher than those in the US general population, especially among the elderly population. Although the results of this study should only be applied to noninstitutionalized individuals, as the issues associated with clinical decision-making in the screening and treatment of the institutionalized population may differ from the noninstitutionalized population in other ways, it is possible that the individuals who respond to surveys, such as the NHIS, may be healthier on an average than the general population. In this study, we adjusted for proxy-reporting in the survival models and reported survival and life expectancies for self-reported health status. Life expectancies estimated from proxy information were similar to the self-reported data. For an individual who is using this calculator is their health status at that time. Our life tables were constructed using the health status at the survey and baseline. In the survey, we do not have information on their future health status and the individual. Incorporating possible changes and trajectories of the individual’s future health status in the life expectancy remained as future research. Lastly, This study aimed to evaluate and construct a life table that can be used as a simple tool to aid clinical decision-making; thus, we only included health status and essential demographic variables in the model. This study did not include specific risk factors that may interact with health status and impact life expectancy estimates, such as smoking status and/or functional limitations. As such, this will be an important area for future research. Despite the limitations mentioned above, this study is a large population-based study using a single question about health status, which is well-validated and easily obtained by physicians or other health care providers. Moreover, self-rated health has been used in numerous population-level studies. It is also a conventional way to address a topic of health status—when is it to be covered in physician-patient communications [30]. Thus, these life tables can provide health care providers a simple tool for assessing life expectancy while adjusting for the health status of their patients.

The National Cancer Institute Surveillance Research Program is developing an online system, the SEER Cancer Survival Calculator, to individually predict cancer patients’ risk of death from cancer and other causes in the US [45, 46]. However, individuals diagnosed with specific cancers are not necessarily representative of the US population at large; thus, using US life tables to represent their general health profile has limitations [47]. The SEER Cancer Survival Calculator tool uses life tables with comorbidities prior to formulating a cancer diagnosis to estimate risk of death because of other causes [45, 46]. However, these life tables are not available for individuals below the age of 65 years. To this end, the life tables using self-reported health developed in this study will be used to estimate the risk of death because of other causes and to provide more accurate estimates of other causes of mortality than general US life tables based solely on demographic characteristics.

## Conclusions

Although US life tables represent the life expectancy of the average person in the general population, they do not fully capture the variation in survival by health status. As shown in this study, health status is strongly associated with life expectancy, especially for younger adults. The life table developed can be helpful by including a patient perspective on their health and be used in conjunction with other predictive models in clinical decision making, particularly for younger adults in poor health, for whom life tables including comorbid conditions are limited.

## Supplementary Information

**Additional file 1.**

## Data Availability

The datasets analyzed during the current study are publicly available from the National Center for Health Statistics, USA (https://www.cdc.gov/nchs/data-linkage/mortality.htm).

## Abbreviations

NHIS: National Health Interview Survey
NCHS: National Center for Health Statistics
SEER: Surveillance Epidemiology and End Results
