# Supplementary material for: Estimating life expectancy adjusted by self-rated health status in the United States: national health interview survey linked to the mortality
Source: BMC Public Health. 2022 Jan 20;22:141. doi: 10.1186/s12889-021-12332-0 (PMC8772174; doi:10.1186/s12889-021-12332-0)
Supplement: Supplementary file 1 — Additional file 1. [file 12889_2021_12332_MOESM1_ESM.docx]

**< Supplementary Materials >**

Estimating life expectancy adjusted by self-rated health status in the United States: National health interview survey linked to the mortality

Hyunsoon Cho, Zhuoqiao Wang, K. Robin Yabroff, Benmei Liu, Timothy McNeel, Eric J. Feuer, Angela B. Mariotto

***Best matching life tables and identification of health-adjusted age:***

The health-adjusted age of white men (women) at age 60 years in *excellent*, *very good*, *good*, *fair* and *poor* health are 51, 55, 58, 64 and 69 years, respectively (53, 55, 58, 64 and 70 in women). Thus, at age 60, survival experiences of white men in *excellent* health is similar to the average white men at age 51 years in the US general population, while the survival experiences of men in *poor* health compares to those of US general population at age 69 years. The decennial 2000 U.S. life tables were used and compared to modelled survival curves up to maximum follow up period providing reliable estimates.

**Supplementary Figure 1**. Estimated survival probabilities for white men (left) and women (right) aged 60 years by health status, compared with the average US white men and women, respectively: An example of identifying health-adjusted age


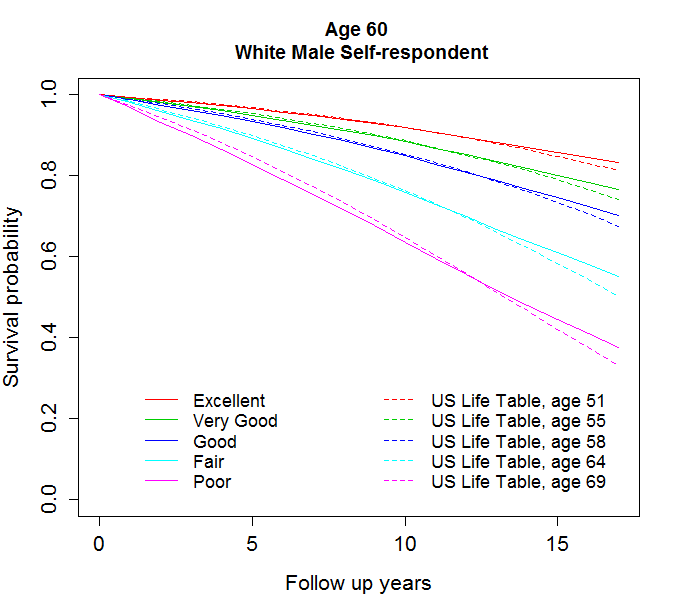

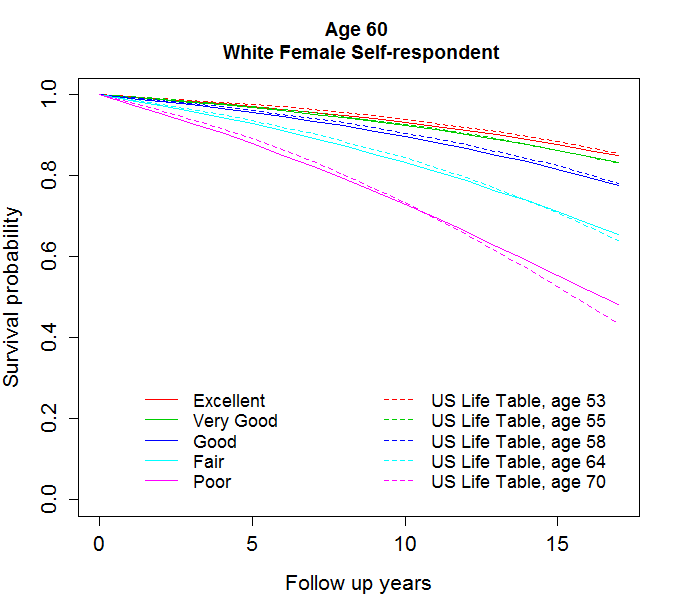


Following the methods detailed in the Mariotto et al. (2013)[1], estimating health-adjusted age was done as follows:

The age adjusted for health status is calculated by searching the US life table that provides the closest survival experience to the estimated life table with a specific chronological age and health status. Let $S_{agp,g}(t|a,r,x)$ the net probability of surviving up to age *a + t* for individuals at age *a* in the age group *agp*, health status *x*, sex *g,* and race *r*, estimated using the model. The term $S_{a,g,r}^{US}(t)$ represent the cumulative probability of surviving up to age *a + t* for a person in the US alive at age *a*, sex *g,* and race *r*, calculated using annual probabilities of death provided in the 2000 US life table. To estimate the health-adjusted age of an individual aged *a* with health status *x*, we calculated the absolute difference between the estimated individual survival and the US survival for a range of ages. The health adjusted age B was the age of the US survival closest to the estimated survival. In mathematical terms, the absolute difference was calculated for a range of ages b as

$$\mathrm{ABSD}\left( b | a,g,r,x \right)=\sum_{t=1}^{17} \{\left\{ \left| S_{agp,g}\left( t | a,r,x \right)-S_{a,g,r}^{US}(t) \right| \right\}$$

The health-adjusted age was given by $B = min\{b, ABSD(b | a, g, r, x) | 50 \leq b \leq110\}$. Although the data allow for a maximum of 21 years of follow-up, we limited the comparisons to 17 years of follow-up because most data estimates have more variability at longer follow-up dates.

***Sensitivity Analysis:***

We compared results from the discrete time proportional hazards models with stratified Cox models and left truncated survival models.

- A stratified Cox model can be used when capturing differences in baseline hazards across age groups but not for the differences in the estimates of a specific health status (possible variabilities in the effect of health status by age, if any, would be included in the baseline hazard estimates). One can also consider a Cox proportional hazards model including age (or age group) as a covariate, where baseline hazards assumed to be the same across all ages.
- When working with a relatively small sample sized study population (or a particular cohort), left-truncated and right-censored survival model with age as a time axis can be a good alternative.

**Supplementary Figure 2.** Comparison of estimated survival curves at age 60 in whites (a) male, (b) female.

Estimated survival from the discrete time proportional hazards model (Discrete), stratified Cox model (Stratified), left truncated model (LT).

**(a)**

**(b)**


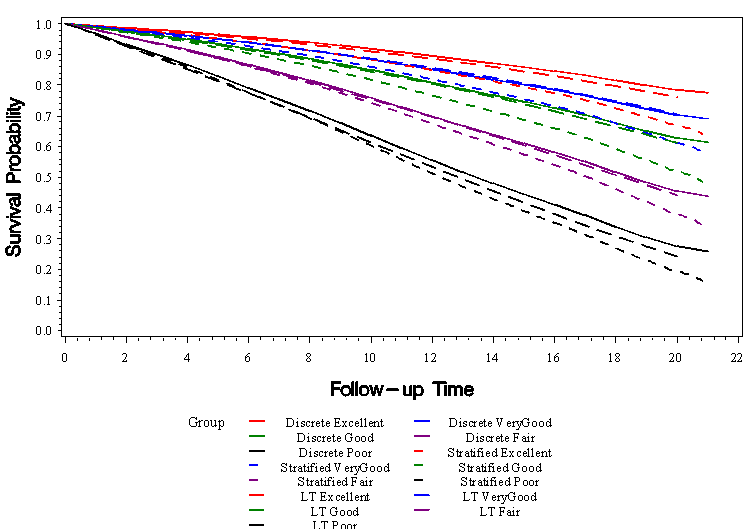

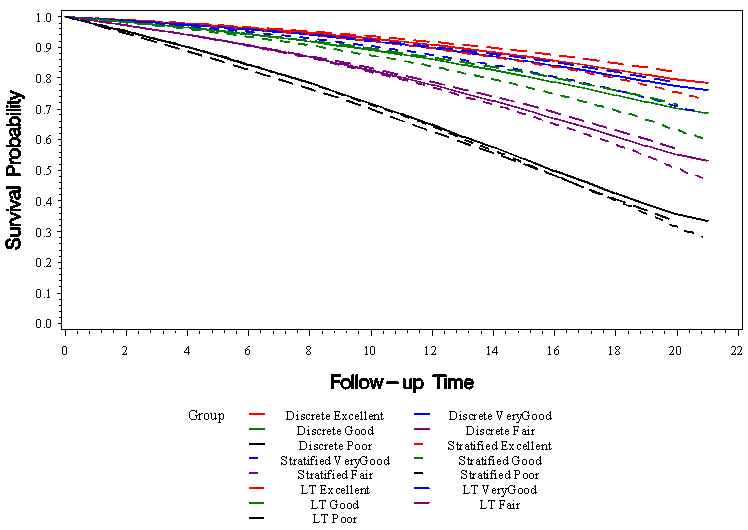


**Supplementary Figure 3.** Hazards ratios for death and 95% confidence intervals by age groups: Proxy reporting versus Self-reporting of health status.


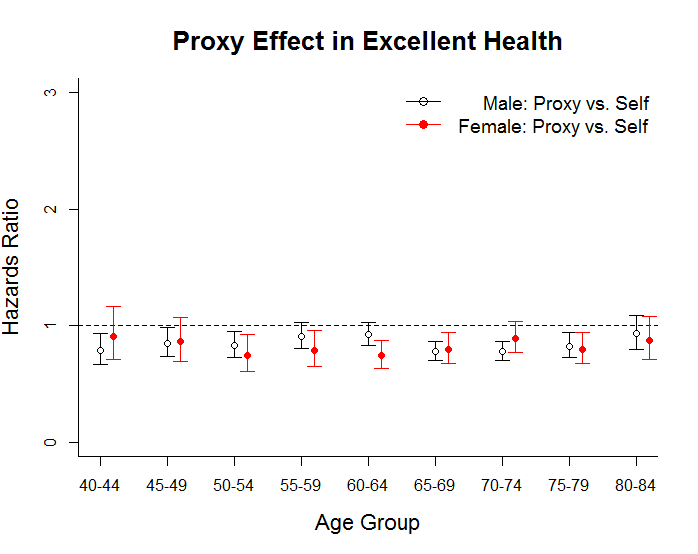

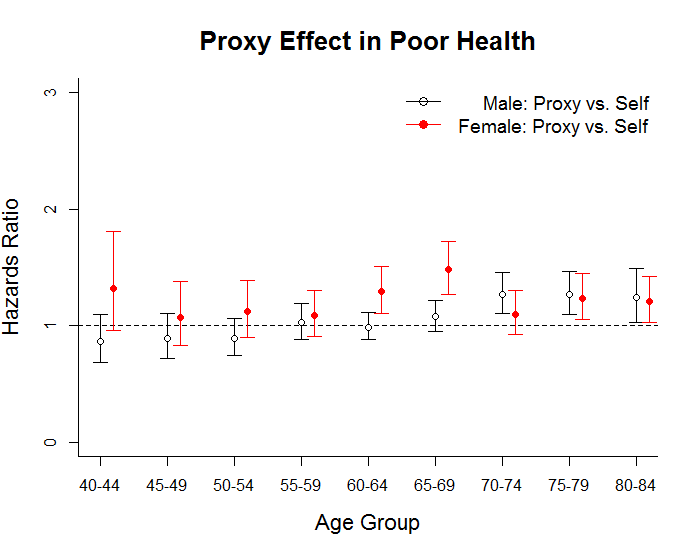


Compared to the self-report, health status reported by other family members (proxy-report) show lower hazards of death in excellent health at all ages and higher hazards of death in poor death at older ages. However, the magnitude is minor and the effects are not significant for some age groups

**Supplementary Table 1.** Parameter Estimates

|  |  |  |  |  |  |  |  |  |  |  |  |  |  |  |  |  |  |  |  |  |  |  |  |  |  |  | |  |
| --- | --- | --- | --- | --- | --- | --- | --- | --- | --- | --- | --- | --- | --- | --- | --- | --- | --- | --- | --- | --- | --- | --- | --- | --- | --- | --- | --- | --- |
|  |  | Age |  | HealthStatus  _VeryGood | | HealthStatus  _Good | | HealthStatus  _Fair | | HealthStatus  _Poor | | NotSelf |  | NotSelf  VeryGood | | NotSelf  Good | | NotSelf  Fair | | NotSelf  Poor | | RACE  _Black | | RACE  _Other | | | Year | |
| SEX | AgeGroup | Est | S.E. | Est | S.E. | Est | S.E. | Est | S.E. | Est | S.E. | Est | S.E. | Est | S.E. | Est | S.E. | Est | S.E. | Est | S.E. | Est | S.E. | Est | S.E. | Est | | S.E. |
| Male | 40-44 | 0.06 | 0.01 | 0.28 | 0.08 | 0.71 | 0.08 | 1.40 | 0.09 | 2.14 | 0.10 | -0.24 | 0.08 | 0.12 | 0.12 | 0.16 | 0.11 | -0.02 | 0.13 | 0.09 | 0.15 | 0.45 | 0.05 | -0.14 | 0.09 | -0.02 | | 0.00 |
|  | 45-49 | 0.08 | 0.01 | 0.29 | 0.07 | 0.71 | 0.07 | 1.32 | 0.07 | 2.06 | 0.08 | -0.16 | 0.07 | 0.05 | 0.10 | 0.02 | 0.10 | -0.01 | 0.11 | 0.05 | 0.13 | 0.36 | 0.04 | -0.32 | 0.08 | -0.02 | | 0.00 |
|  | 50-54 | 0.07 | 0.01 | 0.25 | 0.07 | 0.70 | 0.06 | 1.36 | 0.07 | 1.94 | 0.07 | -0.19 | 0.07 | 0.01 | 0.09 | -0.04 | 0.09 | -0.22 | 0.10 | 0.07 | 0.11 | 0.30 | 0.04 | -0.37 | 0.07 | -0.03 | | 0.00 |
|  | 55-59 | 0.09 | 0.01 | 0.24 | 0.06 | 0.61 | 0.06 | 1.25 | 0.06 | 1.66 | 0.06 | -0.09 | 0.06 | -0.06 | 0.08 | -0.07 | 0.08 | -0.16 | 0.08 | 0.12 | 0.10 | 0.22 | 0.04 | -0.29 | 0.07 | -0.03 | | 0.00 |
|  | 60-64 | 0.09 | 0.01 | 0.37 | 0.05 | 0.65 | 0.04 | 1.18 | 0.05 | 1.67 | 0.05 | -0.08 | 0.06 | -0.10 | 0.07 | -0.06 | 0.07 | -0.01 | 0.08 | 0.07 | 0.08 | 0.13 | 0.03 | -0.46 | 0.07 | -0.03 | | 0.00 |
|  | 65-69 | 0.08 | 0.01 | 0.15 | 0.04 | 0.47 | 0.04 | 0.87 | 0.04 | 1.42 | 0.05 | -0.25 | 0.05 | 0.10 | 0.07 | 0.08 | 0.06 | 0.21 | 0.06 | 0.32 | 0.08 | 0.07 | 0.03 | -0.24 | 0.08 | -0.03 | | 0.00 |
|  | 70-74 | 0.08 | 0.01 | 0.11 | 0.04 | 0.36 | 0.04 | 0.71 | 0.04 | 1.25 | 0.05 | -0.25 | 0.05 | 0.07 | 0.07 | 0.18 | 0.06 | 0.30 | 0.07 | 0.48 | 0.09 | 0.03 | 0.04 | -0.43 | 0.06 | -0.03 | | 0.00 |
|  | 75-59 | 0.07 | 0.01 | 0.15 | 0.04 | 0.39 | 0.04 | 0.61 | 0.04 | 1.13 | 0.05 | -0.19 | 0.07 | 0.02 | 0.08 | 0.10 | 0.07 | 0.36 | 0.08 | 0.43 | 0.10 | -0.06 | 0.04 | -0.42 | 0.07 | -0.02 | | 0.00 |
|  | 80-84 | 0.07 | 0.01 | 0.06 | 0.05 | 0.27 | 0.05 | 0.55 | 0.05 | 0.89 | 0.07 | -0.07 | 0.08 | 0.02 | 0.10 | -0.05 | 0.09 | 0.11 | 0.10 | 0.29 | 0.13 | -0.18 | 0.05 | -0.59 | 0.10 | -0.02 | | 0.00 |
| Female | 40-44 | 0.09 | 0.02 | 0.30 | 0.08 | 0.95 | 0.08 | 1.57 | 0.09 | 2.39 | 0.10 | -0.10 | 0.13 | -0.07 | 0.17 | -0.10 | 0.15 | 0.03 | 0.18 | 0.37 | 0.21 | 0.29 | 0.05 | -0.14 | 0.09 | -0.02 | | 0.01 |
|  | 45-49 | 0.07 | 0.01 | 0.30 | 0.07 | 0.78 | 0.07 | 1.39 | 0.07 | 2.14 | 0.08 | -0.15 | 0.11 | -0.08 | 0.16 | -0.05 | 0.13 | -0.05 | 0.16 | 0.22 | 0.17 | 0.30 | 0.05 | -0.21 | 0.09 | -0.02 | | 0.01 |
|  | 50-54 | 0.09 | 0.01 | 0.21 | 0.07 | 0.64 | 0.06 | 1.26 | 0.07 | 1.96 | 0.07 | -0.29 | 0.11 | 0.09 | 0.15 | 0.10 | 0.13 | 0.19 | 0.14 | 0.41 | 0.15 | 0.21 | 0.05 | -0.34 | 0.09 | -0.03 | | 0.00 |
|  | 55-59 | 0.10 | 0.01 | 0.20 | 0.06 | 0.55 | 0.06 | 1.09 | 0.06 | 1.81 | 0.07 | -0.24 | 0.10 | 0.11 | 0.12 | -0.10 | 0.13 | 0.11 | 0.13 | 0.32 | 0.14 | 0.13 | 0.04 | -0.24 | 0.08 | -0.02 | | 0.00 |
|  | 60-64 | 0.08 | 0.01 | 0.12 | 0.05 | 0.44 | 0.04 | 0.96 | 0.05 | 1.51 | 0.05 | -0.30 | 0.08 | 0.10 | 0.11 | 0.07 | 0.09 | 0.03 | 0.11 | 0.56 | 0.11 | 0.05 | 0.03 | -0.28 | 0.07 | -0.02 | | 0.00 |
|  | 65-69 | 0.08 | 0.01 | 0.17 | 0.04 | 0.47 | 0.04 | 0.89 | 0.04 | 1.44 | 0.05 | -0.22 | 0.09 | 0.03 | 0.10 | 0.06 | 0.10 | 0.15 | 0.11 | 0.62 | 0.12 | 0.03 | 0.03 | -0.33 | 0.07 | -0.02 | | 0.00 |
|  | 70-74 | 0.09 | 0.01 | 0.16 | 0.03 | 0.38 | 0.03 | 0.74 | 0.04 | 1.27 | 0.05 | -0.12 | 0.08 | -0.06 | 0.10 | 0.05 | 0.09 | 0.09 | 0.09 | 0.21 | 0.12 | 0.00 | 0.03 | -0.55 | 0.09 | -0.02 | | 0.00 |
|  | 75-59 | 0.09 | 0.01 | 0.12 | 0.03 | 0.32 | 0.03 | 0.57 | 0.04 | 0.99 | 0.04 | -0.23 | 0.09 | 0.05 | 0.11 | 0.05 | 0.10 | 0.30 | 0.10 | 0.44 | 0.12 | -0.10 | 0.04 | -0.33 | 0.07 | -0.02 | | 0.00 |
|  | 80-84 | 0.08 | 0.01 | 0.07 | 0.04 | 0.22 | 0.04 | 0.51 | 0.04 | 0.75 | 0.05 | -0.14 | 0.11 | -0.02 | 0.13 | 0.12 | 0.12 | 0.11 | 0.13 | 0.32 | 0.14 | -0.16 | 0.04 | -0.46 | 0.10 | -0.02 | | 0.00 |

**Technical Details**

**< Mathematical formulation of the discrete time survival model >**

Suppose $T_{i}$ is the discrete survival time variable of the $i$th subject, $\lambda_{it}$ is a discrete time hazard rate defined as $\lambda_{it}=P\left( T_{i}=t | T_{i}\geq t, \boldsymbol{x}_{\boldsymbol{i}} \right)$, where $\boldsymbol{x}_{\boldsymbol{i}}$ is a covariate vector of $i$th subject for $i=1, \cdots, n$, then the discrete time proportional hazards survival model [2] is given by

$\log\left( -\log\left( 1-\lambda_{ij} \right) \right)=\alpha_{j}+\boldsymbol{\beta}^{\boldsymbol{'}}\boldsymbol{x}_{\boldsymbol{i}}$,

where $j$ is an index for the $j$th interval $j=1, \cdots, J$ , $\alpha_{j}$ is a constant related to baseline hazards in the *j*th interval, $\boldsymbol{\beta}$ is a vector of regression coefficients.

Likelihood is given by $L=\prod_{i} \left[ P(T_{i}=t_{i}) \right]^{\delta_{i}}\left[ P(T_{i}>t_{i}) \right]^{{1-\delta}_{i}}$, where $t_{i}$ is the observed survival of the *i*th subject, $\delta_{i}=1$ if $T_{i}=t_{i}$ is an event time and 0 otherwise, $P\left( T_{i}=t \right)=\lambda_{it}\prod_{j=1}^{t-1} (1-\lambda_{ij})$, $P\left( T_{i}>t \right)=\prod_{j=1}^{t} (1-\lambda_{ij})$. If the data are generated by a continuous-time proportional hazards model then $\boldsymbol{\beta}$ in the model in equation (1) is identical to that of the continuous time proportional hazards model [3].

Survival function is given by

$S\left( t | x_{i} \right)={S_{0}(t)}^{exp\left( \boldsymbol{\beta}^{\boldsymbol{'}}\boldsymbol{x}_{\boldsymbol{i}} \right)}$, where $S_{0}\left( t \right)=\sum_{j\leq t} exp\left( -exp\left( \alpha_{j} \right) \right)$.

For ease of exposition, model is described without survey weights. Implementation of analyses

incorporating survey weights and designs in SAS program is provided below.

**< SAS program used in the paper >**

**Step 1: Data preparation**

Preparing a person year data set and create an indicator variable with 1 indicating the year of death

**data** ExpandedData;

      set NHIS4HALT;

     if missing(NumberOfYears) = **0** then

            do; do INTYEAR = **1** to NumberOfYears;

                        if INTYEAR < NumberOfYears then

                              INTDEATH = **0**;

                        else if INTYEAR = NumberOfYears then

                              INTDEATH = MortStatB;

                        output; end;

end;

else output; **run**;

**Step 2: Model fitting incorporating complex survey sample design**

Use of PROC SURVEYLOGISTIC to fit a proportional hazards model for discrete time survival with complementary log-log link:

%let MAIN = RACE_Black RACE_Other HealthStatus_VeryGood HealthStatus_Good HealthStatus_Fair HealthStatus_Poor YearOffset;

%let INTERACTION = NotSelfVeryGood NotSelfGood NotSelfFair NotSelfPoor;

**proc** **surveylogistic** data=ExpandedData;

      strata STRATA;

      cluster PSU;

      weight MORTWT;

      class INTYEAR / param=glm;

      domain AgeGroupB*SEX;

     model INTDEATH (descending) = INTYEAR AgeA &MAIN. NotSelf &INTERACTION.

/ noint link=cloglog technique=newton xconv=**1e-6** ridging=none CLPARM COVB MAXITER=**200**

**run**;

References:

1. Mariotto AB, Wang Z, Klabunde CN, Cho H, Das B, Feuer EJ: **Life tables adjusted for comorbidity more accurately estimate noncancer survival for recently diagnosed cancer patients**. *Journal of Clinical Epidemiology* 2013, **66**(12):1376-1385.

2. Singer JD, Willett JB: **It’s About Time: Using Discrete-Time Survival Analysis to Study Duration and the Timing of Events**. *Journal of Educational and Behavioral Statistics* 1993, **18**(2):155-195.

3. Prentice RL, Gloeckler LA: **Regression Analysis of Grouped Survival Data with Application to Breast Cancer Data**. *Biometrics* 1978, **34**(1):57-67.
